# Visualization and quantification of injury to the ciliated epithelium using quantitative flow imaging and speckle variance optical coherence tomography

**DOI:** 10.1038/s41598-017-14670-9

**Published:** 2017-11-08

**Authors:** Ute A. Gamm, Brendan K. Huang, Emily K. Mis, Mustafa K. Khokha, Michael A. Choma

**Affiliations:** 1 Yale University, Department of Radiology & Biomedical Imaging, Yale University, 333 Cedar Street, New Haven, CT 06520 USA; 2 Yale University, Department of Biomedical Engineering, Yale University, 55 Prospect Street, New Haven, CT 06511 USA; 3 Yale University, Department of Pediatrics, Yale University, 333 Cedar Street, New Haven, CT 06520 USA; 4 Yale University, Department of Genetics, 333 Cedar St., New Haven, CT 06510 USA; 5 Yale University, Department of Applied Physics, Yale University, 15 Prospect Street, New Haven, CT 06520 USA

## Abstract

Mucociliary flow is an important defense mechanism in the lung to remove inhaled pathogens and pollutants. Disruption of ciliary flow can lead to respiratory infections. Multiple factors, from drugs to disease can cause an alteration in ciliary flow. However, less attention has been given to injury of the ciliated epithelium. In this study, we show how optical coherence tomography (OCT) can be used to investigate injury to the ciliated epithelium in a multi-contrast setting. We used particle tracking velocimetry (PTV-OCT) to investigate the cilia-driven flow field and 3D speckle variance imaging to investigate size and extent of injury caused to the skin of *Xenopus* embryos. Two types of injuries are investigated, focal injury caused by mechanical damage and diffuse injury by a calcium chloride shock. We additionally investigate injury and regeneration of cilia to calcium chloride on *ex vivo* mouse trachea. This work describes how OCT can be used as a tool to investigate injury and regeneration in ciliated epithelium.

## Introduction

Mucociliary flow is an important defense mechanism in the lung to remove inhaled pathogens and pollutants. Reduced ciliary flow can lead to recurrent respiratory infections and a resultant increase in morbidity and mortality. Ciliary flow is known to be affected by exogenous factors such as cigarette smoke^1^ and alcohol^2^ as well as by genetic diseases such as primary ciliary dyskinesia^3,4^ and cystic fibrosis^5,6^. However, risk factors for mucociliary failure in the intensive care unit (ICU) are less well understood. While patients are admitted to the ICU with a broad range of illnesses, it is known that patients with a previously healthy respiratory system are at high risk to develop respiratory infection, especially when mechanical ventilation with intubation is performed^7–9^. We hypothesize that a disruption of mucociliary clearance is one factor that contributes to the increased risk for respiratory infections in patients in the ICU. In a previous study, we investigated cilia-driven fluid flow in mice that had been exposed to hyperoxia (100% FiO2), which is a consequence of administering supplemental oxygen. We found that after 69 h exposure to hyperoxia, cilia-driven fluid flow was significantly reduced^10^. The current study focuses on focal and diffuse injury to the ciliated epithelium. Focal injury can be caused by endotracheal tubes during intubation^11,12^ and diffuse injury to the ciliated respiratory epithelium is typical after smoke inhalation^13^ or aspiration of gastric content^14^. However, there has been limited investigation into the effects of focal and diffuse injury to the ciliated epithelium^15–17^. Such investigation would be greatly facilitated by systematic experimental approaches that pair quantitative imaging methods with experimentally-tractable reduced complexity ciliated systems.

The aim of this study is to use optical coherence tomography (OCT) to visualize and quantify injury to the ciliated epithelium of the *Xenopus* embryo and to *ex vivo* mouse trachea. *Xenopus* embryos express cilia on their surface during their development and have therefore become an important model system of ciliary biology^18^. We use particle tracking velocimetry (PTV) to quantify cilia-driven flow and its changes^19,20^. Additionally, we perform 3D speckle variance imaging^19,21^, a technique to visualize the location of active multiciliated cells and to assess the extent of deciliating injury. Here, deciliation refers to loss of cilia. Injuries are generated either focally by damaging a part of the skin using a microscalpel or diffusely by applying calcium chloride (CaCl_2_), which is a known deciliating agent^22–24^. Both forms of injuries lead to either a focal or diffuse loss of cilia that could be quantified and visualized with OCT. Deciliated mouse tracheas were cultured after diffuse injury and regeneration of the ciliated epithelium and cilia driven flow was monitored. Quantification of cilia-driven fluid flow in combination with visualization of ciliary patches is an important tool to investigate mechanisms of injury and loss of ciliary function in a systematic way.

## Methods

**Figure 1:**
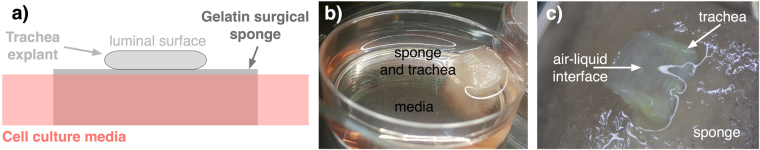
*Ex vivo* tracheal explant culture. (**a**) Schematic of culture setup. (**b**) Photograph of petri dish housing a gelatin sponge upon which a trachea is placed. (**c**) Close-up photograph of trachea on gelatin sponge.

All animal work was done in accordance with relevant guidelines and regulations and was approved by the Institutional Animal Care & Use Committee (IACUC) of the Yale School of Medicine, New Haven, CT, USA. *Xenopus tropicalis* embryos of Nieuwekoop and Faber (NF) stage 33–36 and 36–40 were used for experiments to investigate focal (n = 7) and diffuse injury (n = 6), respectively. Each embryo was kept in 1/9x Modified Ringer (MR) solution and anesthetized with benzocaine before the experiments. Focal injury was caused by a scalpel leaving a superficial injury of approximately 0.4 mm × 0.4 mm in size. Diffuse injury was created by a CaCl_2_-shock through application of a solution of 75 mM CaCl_2_ and 0.02% Tergitol-type NP40 (nonyl phenoxypolyethoxylethanol) in 1/9x MR solution^24^, which largely deciliated the skin of the embryos. Embryos were imaged before and immediately after focal or diffuse injury. Tracheas of 7 mice (C57BL/6, age 2–3 weeks**)** were excised within 30 min after sacrifice and kept in warm DMEM/F12 medium until the experiment. Incubation in 75 mM CaCl_2_ solution for 5 min generated diffuse deciliation in mouse trachea. Following the experiments, tracheas were cultured on a gelatin surgifoam sponge (Fig. 1) in DMEM/F12 medium with 12 mM HEPES buffer and Anti-Anti (Antibiotic-Antimycotic) solution for 8 days to allow cilia to regenerate^17,25^. Imaging was performed before and immediately after CaCl_2_-shock as well as 8 days later.

Imaging was performed using a spectral domain OCT system (Telesto, Thorlabs) with 1325 nm center wavelength. OCT-based particle tracking velocimetry (PTV-OCT) was performed using polystyrene microspheres (Bangslabs, In, USA) of 10 µm diameter. The movies were analyzed with an in-house scripted MATLAB code as described previously^20^. In short, particle tracking analysis consists of 4 steps; 1) Image segmentation distinguishes flowing particles from the immobile parts of the image within a chosen region of interest, 2) Particle identification identifies clusters of pixels as particles, 3) Particle localization determines the coordinates of the center of mass of the particle and 4) Particle pairing matches particles of the subsequent frame which allow the calculation of the flow velocity vectors. For frog embryos the region of interest was chosen to be along the entire extent of the animal and up to 1 mm above it, allowing for a visualization of the whole flow field around the animal. Average flow speeds were calculated from a tangent line along the surface of the embryo. For mouse tracheas, the region of interest was chosen to capture the most superficial tracer beads at approximately 100–150 µm height above the ciliated surface. Average flow speeds were calculated from the vector flow field within the region of interest. Choosing a slimmer region of interest in mice was necessary to avoid including stationary particles into the analysis and to reduce computation time. Computation time was longer in mice than in frog embryos due to a larger number of tracer beads used. Three movies of 100 frames were collected in each experiment. The resolution of the OCT system is not high enough to resolve individual cilia, but nonetheless actively beating cilia on multi-ciliated cells can be collectively visualized by analyzing time-varying speckle patterns^19^. In order to generate 3D rendered speckle variance images, we generated 30 2D images at 81 individual OCT planes, each separated by 10 μm, over an entire ciliated surface. We then calculated the speckle variance over these 30 images and stacked the speckle variance images of all planes to create a 3D rendered image by using ImageJ. Speckle variance enhances the contrast of moving things which results in a bright presentation of moving cilia.

## Results

**Figure 2:**
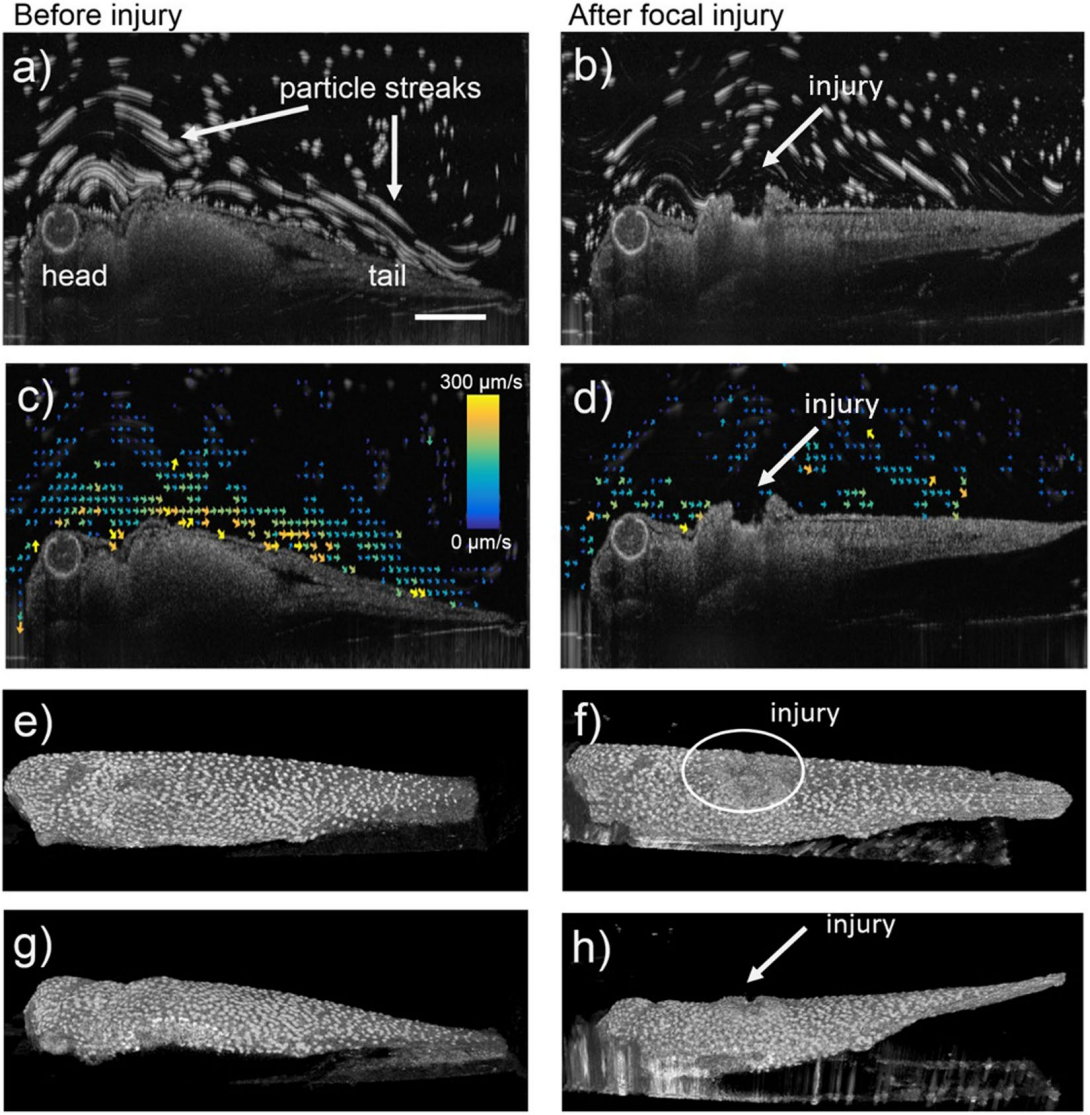
Imaging of ciliated *Xenopus* embryo skin before (**a**,**c**,**e**,**g**) and after focal injury (**b**,**d**,**f**,**h**). (**a,b**) Maximum intensity projection of a 100-frame video allows the visualization of tracer particles as particle streaks. The injury is visible as indentation in the embryos surface (marked with an arrow in b). (**c,d**) Vector flow field quantification of cilia-driven flow. The vector flow field is disrupted around location of injury (panel d). (**e–h**) 3D rendered speckle variance images visualize beating ciliary patches on embryo surface (panels e and f- view from top; panels g and h- view from side). The injury is visible as area with low speckle variance (white circle) in panel f and as indentation in panel h. The scale bar in panel a measures 0.5 mm.

We created a focal injury to the ciliated epidermis of 7 *Xenopus* embryos and imaged the cilia-driven flow field as well as the speckle variance to visualize the location of multiciliated cells with actively beating cilia. Figure 2 displays one embryo before (left column) and after focal injury (right column). Figure 2a and b display maximum projection images of a 100 frame video, which was used to perform particle tracking velocimetry (supplemental videos 1 and video 2). The particles are visible as particle streaks along the surface of the embryo. Figure 2c and d display the vector flow field created through PTV. At the location of the injury (Fig. 2b and d), flow is stagnant due to the lack of cilia and potentially due to an altered geometry of the surface. PTV analysis showed that flow speed in image planes where the injury is visible was reduced by ~20% on average over the 7 embryos (data not shown). The injury was visualized with speckle variance imaging, which enhances the image contrast of moving objects such as beating cilia. Consequently, the embryo surface appears spotted due to the relatively sparse distribution of multiciliated cells (Fig. 2e and g and supplemental video 3) and at the location of injury where cilia are absent a lack in speckle variance is visible (Fig. 2f and h and supplemental video 4).

**Figure 3:**
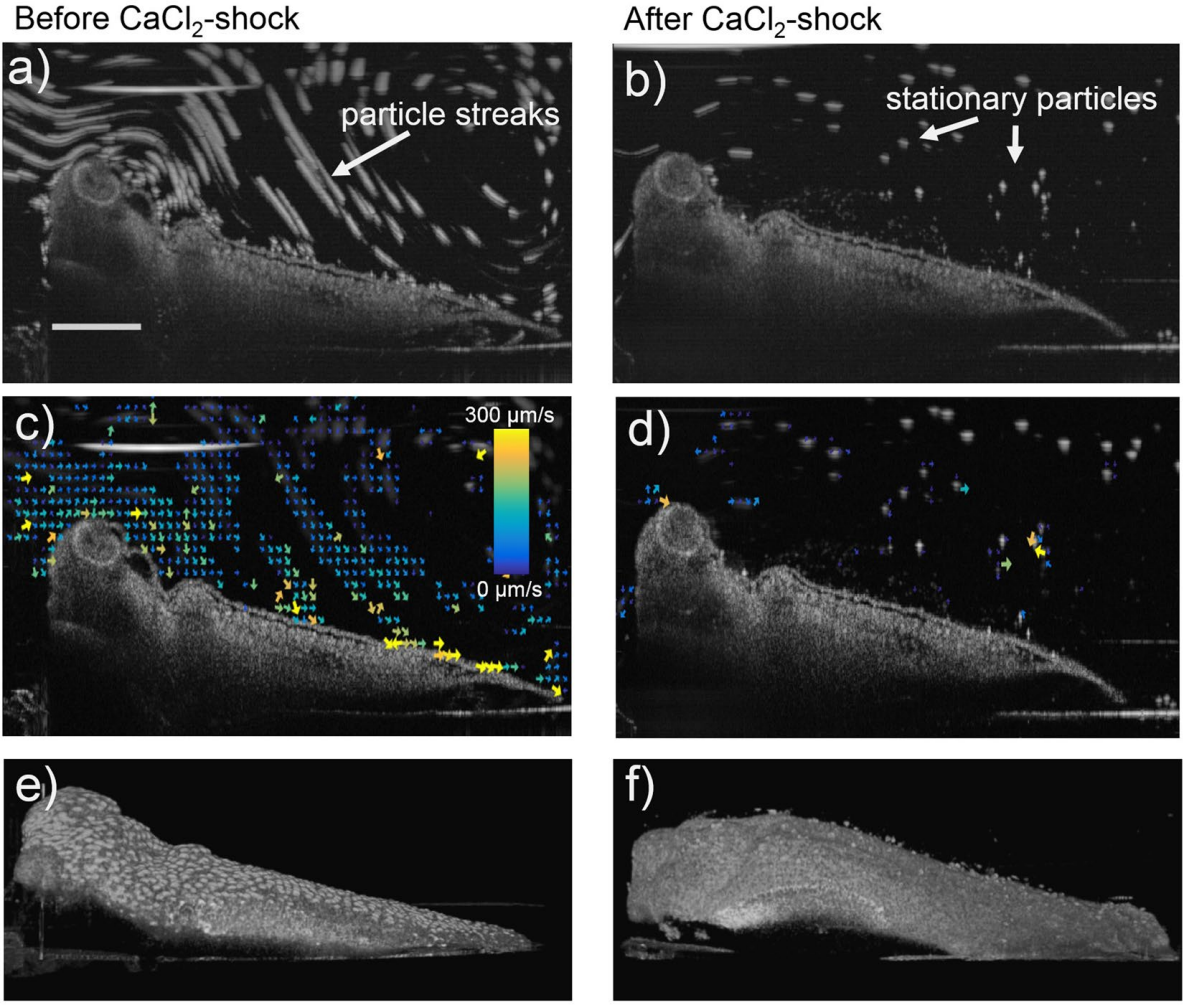
Imaging of ciliated frog embryo skin before (**a**,**c**,**e**,**g**) and after CaCl_2_-shock (**b**,**d**,**f**,**h**). (**a,b**) Maximum intensity projection of a 100-frame video allows the visualization of flowing tracer particles as particle streaks. After application of CaCl_2_ flow is disrupted along the whole embryo surface and tracer particles appear stationary. (**c,d**) Vector flow field of cilia-driven flow. The vector field is non-existent after application of CaCl_2_ (panel d). (**e,f**) 3D rendered speckle variance imaging. After CaCl_2_-induced loss of cilia, the surface appears smooth with uniform grey intensity (panel f). The scale bar in panel a measures 0.5 mm.

**Figure 4:**
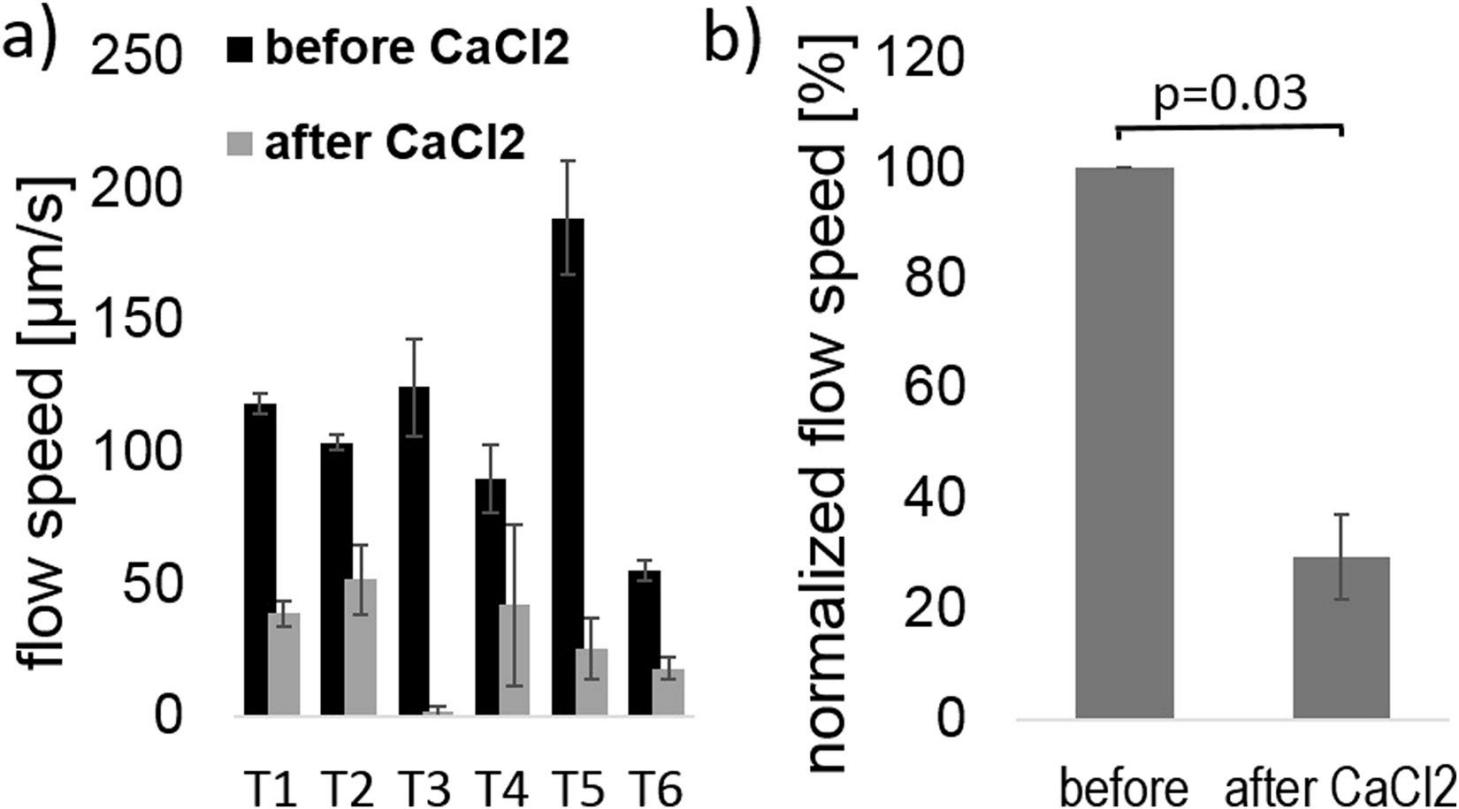
(**a**) PTV-OCT results before and after application of CaCl_2_ to 6 *Xenopus* embryos. T1-T6 describes each individual animal. (**b**) Percentage decrease in flow speed after CaCl_2_ application. Error bars depict standard error. Wilcoxon signed rank test shows a significant decrease in flow speed (p = 0.03).

Figure 3 shows the results of PTV and speckle variance imaging before (a,c,e) and after (b,d,f) partial deciliation of the embryo skin by CaCl_2_. From the maximum projection image in Fig. 3b) it can be concluded that cilia-driven flow was stopped after deciliation because particles do not appear as streaks any more but rather as stationary particles (also note supplemental videos 5 and 6 before and after application of CaCl_2_). Figure 3d shows that PTV analysis failed to create a flow field, due to the absence of flow. In some embryos a few ciliary patches remained in the skin and remained generating flow. Speckle variance imaging in Fig. 3e and f as well as supplemental videos 7 and 8 show near-complete deciliation after application of CaCl_2_. Residual ciliation was capable of creating a flow that is quantifiable by PTV. The CaCl_2_-shock causes an instant loss of cilia and we were able to monitor this event under the OCT. Residual cilia can be seen floating within approximately 200 µm above the body of the animal in Fig. 3b and d as small grey dots. Deciliation was performed on 6 frog embryos. The results are summed up in Fig. 4 where flow speeds of individual animals before and after CaCl_2_ application (Fig. 4a) and the percentage decrease of flow speed (Fig. 4b) are shown. Error bars show the standard error that resulted from analyzing multiple videos per animal in Fig. 4a and propagation of uncertainty in Fig. 4b. Statistical testing (Wilcoxon signed-rank test) resulted in a p-value of 0.03 and therefore a significant decrease in cilia-driven flow after application of CaCl_2_. Overall, application of CaCl2 deciliated *Xenopus* embryos to a large extent and its effect on the embryo can be visualized and quantified by PTV and 3D rendered speckle-variance imaging using OCT.

**Figure 5:**
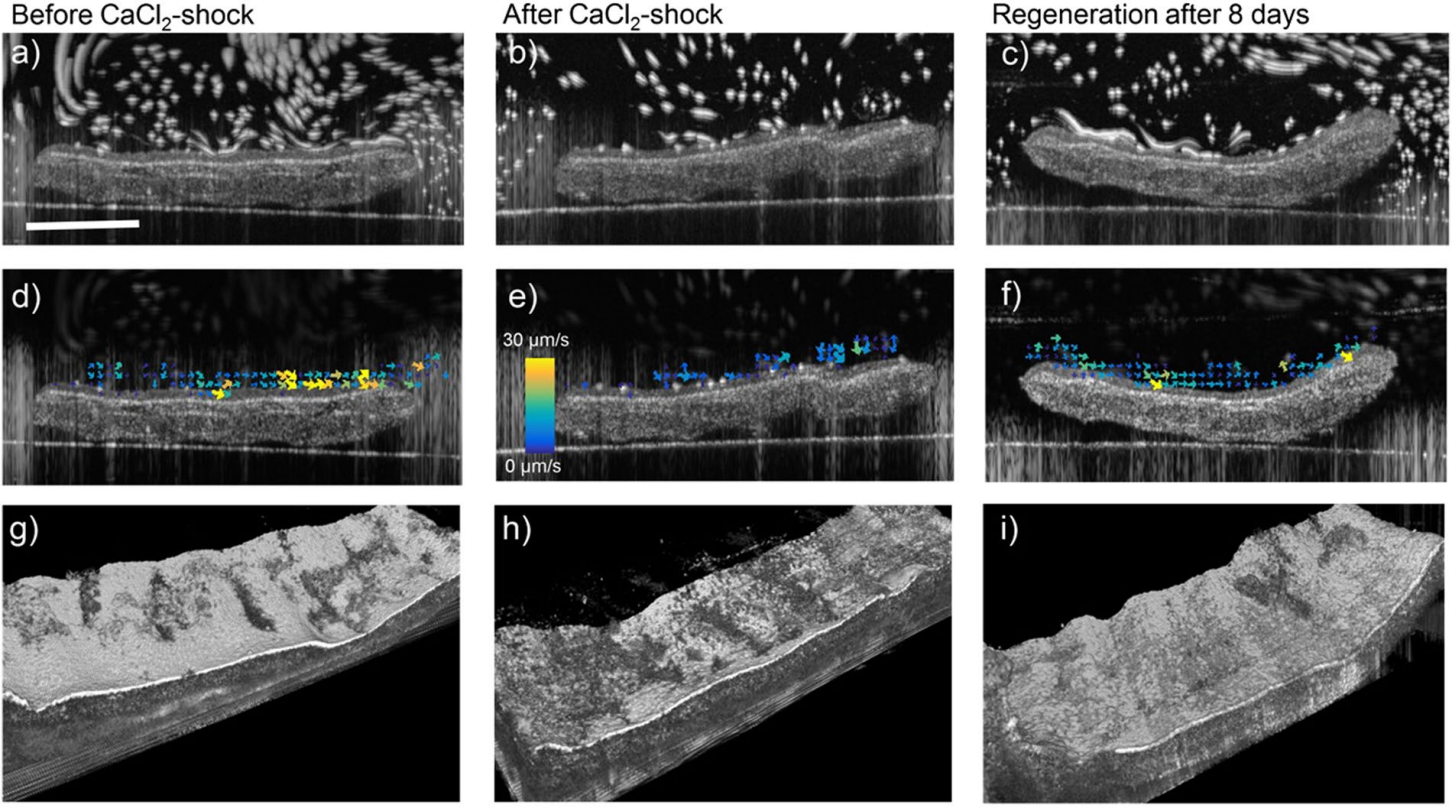
OCT Imaging of mouse trachea before (**a**,**d**,**g**), after CaCl_2_-shock (**b**,**e**,**h**) and after regeneration 8 days later (**c**,**f**,**i**). (**a–c**) Maximum intensity projection of a 200-frame video allows the visualization of flowing tracer particles as particle streaks. After application of CaCl_2_ flow is disrupted along the whole epithelium and tracer particles appear stationary. Flow is restored after 8 days of *ex vivo* culture. (**d–f**) Vector flow field of cilia-driven flow, gained by PTV analysis. The vector flow field shows very small and undirected movement of tracer beads after application of CaCl_2_ and complete restoration after regeneration. (**g–i**) 3D rendered speckle variance images visualize beating cilia as area of high image intensity. After CaCl_2_-induced loss of beating cilia, the surface appears spotted with an increase of low variance area h). After cilia restore, the surface appears smooth with high speckle variance again i). The scale bar in a) measures 0.5 mm.

We investigated injury and regeneration in a mammalian animal model by applying CaCl_2_ to *ex vivo* mouse tracheas. Figure 5 shows similar to Figs 1 and 2 cross sectional and vector flow images, as well as 3D rendered speckle variance images before and after CaCl_2_ application and after and 8 day regeneration period. The CaCl_2_-shock also deciliated large parts of the tracheal epithelium which resulted in a disruption of cilia driven flow. PTV analysis was only performed within a region of interest close to the tracheal surface which is displayed in a narrower vector flow field in Fig. 5d–f. It can be seen that after CaCl_2_ the vector flow is slow and undirected. After regeneration of cilia after 8 days, cilia driven flow has recovered and the vector flow field appears similar to the field in Fig. 5d before CaCl_2_ application. The deciliating effect of CaCl_2_ is visualized in Fig. 5(g–i). Mouse tracheas are more densely ciliated than frog embryos which results in an even layer of high speckle contrast in Fig. 5g). After CaCl_2_ application (Fig. 5h)) a large part of the surface is deciliated and thus speckle contrast is lost, which appears as dark spots. Regenerated epithelium is densely ciliated again and the speckle contrast appears is high and evenly distributed over the surface.

**Figure 6:**
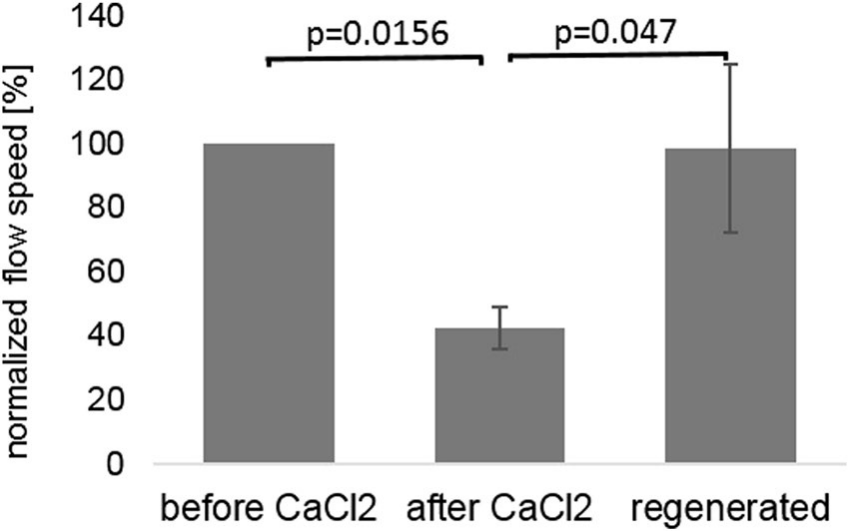
PTV results before and after application of CaCl_2_ to 7 mouse tracheas and after regeneration 8 days later. P-values were calculated using the Wilcoxon signed rank test.

Results of PTV analysis on 7 tracheas are displayed in Fig. 6. After CaCl_2_ application, flow speeds dropped in average down to 40% of their initial values and were restored to their initial values after a period of eight days which allowed the tissue to recover and regenerate cilia.

## Discussion

This article describes how OCT can be used in a multi-contrast fashion to visualize and quantify injury to the ciliated epithelium of *Xenopus* embryos and *ex vivo* to the ciliated respiratory epithelium of mice, where we also monitored regeneration. Focal injury as well as diffuse injury could be visualized very well with speckle variance imaging, and a decrease in flow speed was quantified through OCT-PTV. Limitation of the method is that both speckle variance imaging and OCT-PTV analysis take a considerable amount of computational power and time, which only allow post-processing and not real-time analysis.

Deciliation of frog embryos using a CaCl_2_-shock happened within a few seconds after application. However, in mouse tracheas the effect of deciliation became only visible after a few minutes and we chose to incubate the tracheas for 5 minutes in CaCl_2_ solution. One hypothesis to explain the longer incubation time might be the higher density of cilia on a mouse trachea. A higher concentration of calcium chloride might have caused a more immediate effect. Another hypothesis is that a thin film of mucus that was not detectable under the OCT could have protected the trachea for a few minutes from the damaging effect of calcium chloride.

In the future, our group aims to use the combination of quantitative flow analysis with qualitative visualization of ciliary pattern to correlate specific patterns of injury with flow patterns to improve fundamental understanding of ciliary function. Our approach in *Xenopus* can be used in a relatively straightforward manner to study general questions such as the relationship between patterns of ciliation after injury and the flow field generated by the ciliated surface. Our approach in mouse can be used to study specific factors that may lead to deciliation in the ICU (e.g. low pH fluid, hyperoxia) and drugs that can be prophylactically given to mitigate damage caused by these exposures. We also expect that the approach presented here can be extended to patient-derived ciliated cultures. In addition to these lab-based future directions, data generated about injury and regeneration to ciliated surfaces provides further motivation to the ongoing development of endoscopic/bronchoscopic *in vivo* OCT technologies (e.g.^26,27^) for the potential longitudinal imaging of the injured tracheal mucosa in the ICU. In conclusion, utilizing multi-contrast OCT allows a multi-faceted investigation of injury to the ciliated epithelium by combining (a) PTV for a quantitative flow analysis with (b) 3D rendered speckle-variance imaging for visualization of ciliary patches.

## Electronic supplementary material

OCT-PTV before focal injury

OCT-PTV after focal injury

3D rendered speckle variance imaging before focal injury

3D rendered speckle variance imaging after focal injury

OCT-PTV before diffuse injury

OCT-PTV after diffuse injury

3D rendered speckle variance imaging before diffuse injury

3D rendered speckle variance imaging after diffuse injury
